# Hemoperfusion Combined With Continuous Renal Replacement Therapy in the Management of ARDS COVID‐19 Patients: A Quasi‐Experimental Study

**DOI:** 10.1002/hsr2.70571

**Published:** 2025-04-01

**Authors:** Seyed MohammadReza Hashemian, Ameneh Jafari, Batoul Khoundabi, Hamidreza Jamaati, Payam Rahimi

**Affiliations:** ^1^ Chronic Respiratory Diseases Research Center, National Research Institute of Tuberculosis and Lung Diseases (NRITLD) Shahid Beheshti University of Medical Sciences Tehran Iran; ^2^ Iran Helal Institute of Applied‐Science and Technology Red Crescent Society of Iran Tehran Iran; ^3^ Department of Anesthesiology and Reanimation, Bakırköy Dr. Sadi Konuk Training and Research Hospital University of Health Sciences Istanbul Turkey

**Keywords:** acute respiratory distress syndrome, ARDS, continuous renal replacement therapy, COVID‐19, CRRT, cytokine, hemoperfusion, mortality, procalcitonin

## Abstract

**Background and Aims:**

Critically ill patients in COVID‐19 to the intensive care unit (ICU) may develop multiple organ dysfunction syndrome, with some requiring extracorporeal organ support. This study aimed to assess the effects of combined CytoSorb hemoperfusion (HP) and continuous renal replacement therapy (CRRT) on the improvement of the multiorgan failure of patients with COVID‐19.

**Methods:**

Fifty‐six patients hospitalized in the ICU with a confirmed diagnosis of COVID‐19 were included in this quasi‐experimental study. All the patients had acute respiratory distress syndrome (ARDS). They were treated with 1–4 sessions of HP therapy.

**Results:**

Serum Interleukin‐6 (IL6), C‐reactive protein (CRP), d‐dimer, procalcitonin (PCT), Neutrophil gelatinase‐associated lipocalin (NGAL), ferritin, and bilirubin levels were decreased, while the concentration of albumin was significantly increased after HP/CRRT (*p* < 0.05). No significant differences were observed in O2 saturation (Sao2) and creatinine levels.

**Conclusion:**

Combined HP and CRRT hold promise as a potential intervention for severe COVID‐19 cases with multiple organ dysfunction, leading to improved clinical outcomes.

## 1 Introduction

As of January 7, 2024, a total of 774,075,242 confirmed cases of COVID‐19 including 7,012,986 deaths have been reported to the World Health Organization (WHO) [1], indicating the significant impact of disease on global healthcare systems. COVID‐19 has presented a wide spectrum of symptoms in infected patients, varying from mild to severe [2, 3]. Severe cases of COVID‐19 often rise to acute respiratory distress syndrome (ARDS), acute kidney injury (AKI), and multiorgan dysfunction, necessitating intensive care unit (ICU) admission and Extracorporeal organ support [4]. This severe condition is characterized by an excessive release of pro‐inflammatory cytokines, such as interleukin (IL)‐1β and IL‐6, which orchestrate hyperinflammatory state [5]. Uncontrolled overproduction of these cytokines results in the progression of multiple organ failure, including ARDS and AKI, and is associated with a high mortality rate. Numerous studies have demonstrated a direct correlation between the mortality rate of COVID‐19 patients and the levels of both pro‐ and anti‐inflammatory cytokines [6]. Consequently, the administration of immunomodulatory agents holds promise in regulating this hyperinflammatory response and potentially enhancing clinical outcomes [7, 8]. Furthermore, the implementation of extracorporeal therapies may be beneficial in managing the overwhelming inflammatory storm in patients [9].

In this context, hemoperfusion (HP) and continuous renal replacement therapy (CRRT) have the potential to effectively remove inflammatory cytokines from the bloodstream, modulate immune function, and positively impact patient outcomes [9, 10]. CRRT, a type of blood purification technology, has the capacity to treat ill COVID‐19 patients by eliminating harmful toxins and stabilizing their metabolic and hemodynamic conditions. While CRRT has become a standard supportive intervention for managing acute kidney injury in critically ill patients, conventional CRRT alone may not effectively address the complex pathophysiological changes observed in COVID‐19 patients [11, 12]. HP utilizes specific adsorbents to remove inflammatory mediators from the bloodstream have been advocated as a promising adjunctive therapy in the management of critically ill patients with various inflammatory conditions. AKI is a common complication in COVID‐19 patients, significantly affecting their prognosis [13]. Studies from the Italian group of intensivists and nephrologists have demonstrated the potential benefits of HP in patients with AKI, both with and without COVID‐19 infection [14, 15]. These findings highlight the potential of HP to enhance kidney function and improve clinical outcomes in critically ill patients. Given the pathophysiological features of COVID‐19, the combination of these two modalities, HP and CRRT, could potentially mitigate the overwhelming inflammatory response, prevent multiorgan dysfunction, and ultimately enhancing patient survival [16, 17].

This study aims to evaluate the effectiveness of combining Hemoperfusion with CRRT in COVID‐19 patients by analyzing laboratory characteristics before and after treatment, as well as the follow‐up data over specific time intervals. The analysis will focus on key markers related to inflammation, coagulation, kidney function, liver function, and protein status. The aim is to shed light on how this combined treatment strategy affects the chosen indicators and offer insights into its potential advantages in treating severe COVID‐19 patients.

## 2 Materials and Methods

### 2.1 Study Design and Participants

This study was carried out at Masih Daneshvari Hospital, Shahid Beheshti University of Medical Sciences, after the approval of the study protocol by the Clinical Research Ethics Committee (IR.SBMU.NRITLD.REC.1399.011). Informed consent was obtained from all study participants or a family member, such as a parent, sibling, or spouse, as documented. A total of 56 patients diagnosed with COVID‐19 were included in this study during the fall and winter of 2021 in Masih Daneshvari Hospital as a referral education center. Of which, 34 (60.7%) were male, and 22 (39.3%) were female. These patients required mechanical ventilation (MV) and exhibited a partial pressure of arterial oxygen/fraction of inspired oxygen ratio (PaO2/FiO2) below 150, with a confirmed diagnosis of COVID‐19 using reverse transcription polymerase chain reaction (RT‐PCR). The initial blood sample was collected immediately upon the patient's admission to the ICU, before any treatment was administered. Inclusion criteria contains patient who had ARDS and the complete data file and consent to enter in study. Direct and surrogate consent was allowed. Failure to meet inclusion criteria was considered as exclusion criteria:

All patients were supported by HP (Cytosorb 300®, Cytosorbents USA) and CRRT (Infomed HF440®, Switzerland) devices. In AKI‐positive patients, HP was used in combination with CRRT. In AKI‐negative patients, HP was used alone. HP therapy was initiated within 24 h of ICU admission for all patients. This timing was selected based on previous studies highlighting the critical impact of early HP intervention. All clinical data were recorded and standardized in a spreadsheet by two critical care physicians who were unaware of the study's purpose.

### 2.2 Medical Care in ICU

All patients received standard care in the ICU, which included support with ventilators, nutrition through enteral or parenteral methods, replacement of fluids, correction of water‐electrolyte imbalances, and management of body temperature. The treatment regimen involved administering hydroxychloroquine sulfate at a dose of 400 mg twice a day on the first day, followed by 200 mg twice a day [18]. To prevent pulmonary damage, patients were given atazanavir and ritonavir once daily for a period of 7–14 days. Bacterial and atypical pneumonia were treated with vancomycin (a loading dose of 25 mg/kg, followed by 15 mg/kg twice a day), imipenem (500 mg every 6 h) IV infusion, and azithromycin (500 mg daily for 3 days). Norepinephrine was administered intravenously at a dosage ranging from 0.5 to 2.5 mcg/kg/min to enhance blood perfusion and cardiac function. The effectiveness of the treatment was assessed by analyzing various blood parameters, such as arterial blood gas measurements, PaO2 and O2 saturation, hemodynamic indicators (central venous oxygen saturation (ScvO2) and oxygen delivery (DO2)), plasma levels of IL6, albumin (ALB), lactate dehydrogenase (LDH), d‐dimer, as well as kidney function markers (blood urea nitrogen (BUN) and serum creatinine (SCr)). A favorable treatment outcome was defined as a significant improvement in the severity of ARDS, demonstrated by an increase in the PaO2/FiO2 ratio, O2 saturation, DO2, ScvO2, improved renal function as well as reduced levels of inflammatory cytokines and norepinephrine demand, ultimately leading to improved survival. The survival time was calculated from the time of ICU admission to the patient's discharge from the hospital.

### 2.3 Blood Purification

To improve oxygen saturation and reduce the levels of pro‐inflammatory cytokines, HP therapy combined with CRRT was initiated. The presence of elevated IL‐6 levels in ARDS patients with normal kidney function indicated its involvement in severe lung injury. The number of HP sessions was determined based on the clinical condition and response of each patient. Patients who exhibited ongoing inflammation and cytokine levels received additional HP sessions. The adsorber was routinely replaced every 12 h during the 24‐h period of CRRT due to the saturation of the adsorptive sites, following aseptic techniques. To prevent clotting during each blood purification technique, a continuous infusion of heparin was administered at a dosage ranging from 5 to 20 units/kg/h. The therapeutic level of anticoagulation was defined by an activated partial thromboplastin time ranging between 1.5 and 2 times the normal value.

### 2.4 Data Collection

The sequential organ failure assessment (SOFA) score and APACHE II score were calculated for each patient upon their admission to the ICU. A commercially available ELISA kit (Antibody Shop, Gentofte) was used to measure serum Neutrophil gelatinase‐associated lipocalin (NGAL) levels. Blood samples treated with ethylene diamine tetra acetic acid (EDTA) were collected before and after the therapeutic procedures in sterile (endotoxin‐free) tubes. Samples intended for cytokine detection were kept on ice and promptly transported to the laboratory. Plasma was separated by centrifugation (2000 *g*/10 min/at 4°C) and stored in 300 μL portions at −70°C until analysis. The concentrations of IL‐6 in the serum were determined by ELISA using Quantikine kits according to the manufacturer's instructions. ScvO2 and DO2 were measured using pulse contour cardiac output (PiCCO) via a central venous catheter and an arterial line. Measuring DO2 relied on parameters such as hemoglobin/hematocrit levels, cardiac output, and the saturation of hemoglobin molecules. Blood samples were also analyzed simultaneously for acute phase reactants (ferritin and procalcitonin (PCT)), kidney function indicators (BUN, SCr), LDH. ALB, d‐dimer, and blood gas factors (PaO2, O2 sat) using an automated biochemical analyzer, along with the determination of cytokine levels.

### 2.5 Statistical Analysis

Data were analyzed using SPSS software version 26 (IBM Inc.) in addition to GraphPad software version 9 (Dotmatics). Categorical variables were presented using numbers and percentages, and quantitative data in normal distribution were described by mean and standard deviation (Mean ± SD) and in the non‐normal state, using median and IQR (Interquartile Range), respectively. Statistical tables and graphs were used according to the type of variables. The normality of quantitative variables was checked using the Kolmogorov–Smirnov test. The comparison of quantitative variables in two paired groups was done using a dependent *t*‐test. Repeated measurement analysis was applied to compare the amount of variables through time. Effect size amounts are reported by Cohen *d* index to measure the strength of the relationship between two variables in a population or a sample‐based estimate of that quantity. A commonly used interpretation is to refer to effect sizes as small (*d* = 0.2), medium (*d* = 0.5), and large (*d* = 0.8) based on benchmarks suggested by Cohen [19]. The significance level of all tests was considered two‐sided 0.05.

## 3 Results

A total of 56 patients diagnosed with COVID‐19 were studied, including 34 men (60.7%) and 22 women (39.3%). The average age of the patients is 60.8 ± 15.1 years. Blood pressure and diabetes were the most common underlying diseases in 14 (25.0%) and 13 (23.2%) patients, respectively. Nineteen patients (33.9%) underwent mechanical ventilation (MV). The average duration of hospitalization of patients in the intensive care unit and the duration of MV is 11.5 ± 7.3 and 4.0 ± 3.5 days, respectively (Table 1). 36 (64.7%) were treated with cytosorb hemoperfusion at least one session, 13 (23.5%) and 5 (8.8%) patients two and three sessions, and only 2 (2.9%) patients treated with four session cytosorb hemoperfusion.

**TABLE 1:** Demographic and underlying variables of patients.

Item	*n* (%)/Mean ± SD
Gender (Male)	34 (60.7)
Age (Year)	60.8 ± 15.1
ICU Stay time (Day)	11.5 ± 7.3
Ward Stay Time (Day)	5.6 ± 3.9
Mechanical Ventilation Time (Days)	4.0 ± 3.5
Extubation time (Days)	2.0 ± 1.6
Mortality (Expired)	23 (41.1)
Mechanical Ventilation (Yes)	19 (33.9)
Intubation (Yes)	15 (26.7)
Creatinine	1.1 ± 1.0
APACHE	48.9 ± 5.9
One Session of Cytosorb	*N* = 36 (64.7%)
Two Sessions of Cytosorb	*N* = 13 (23.5%)
Three Sessions of Cytosorb	*N* = 5 (8.8%)
Four Sessions of Cytosorb	*N* = 2 (2.9%)
Underlying Disease (Diabetes)	13 (23.2)
Underlying Disease (HTN)	14 (25.0)

*Note:* SD, standard deviation

**FIGURE 1:**
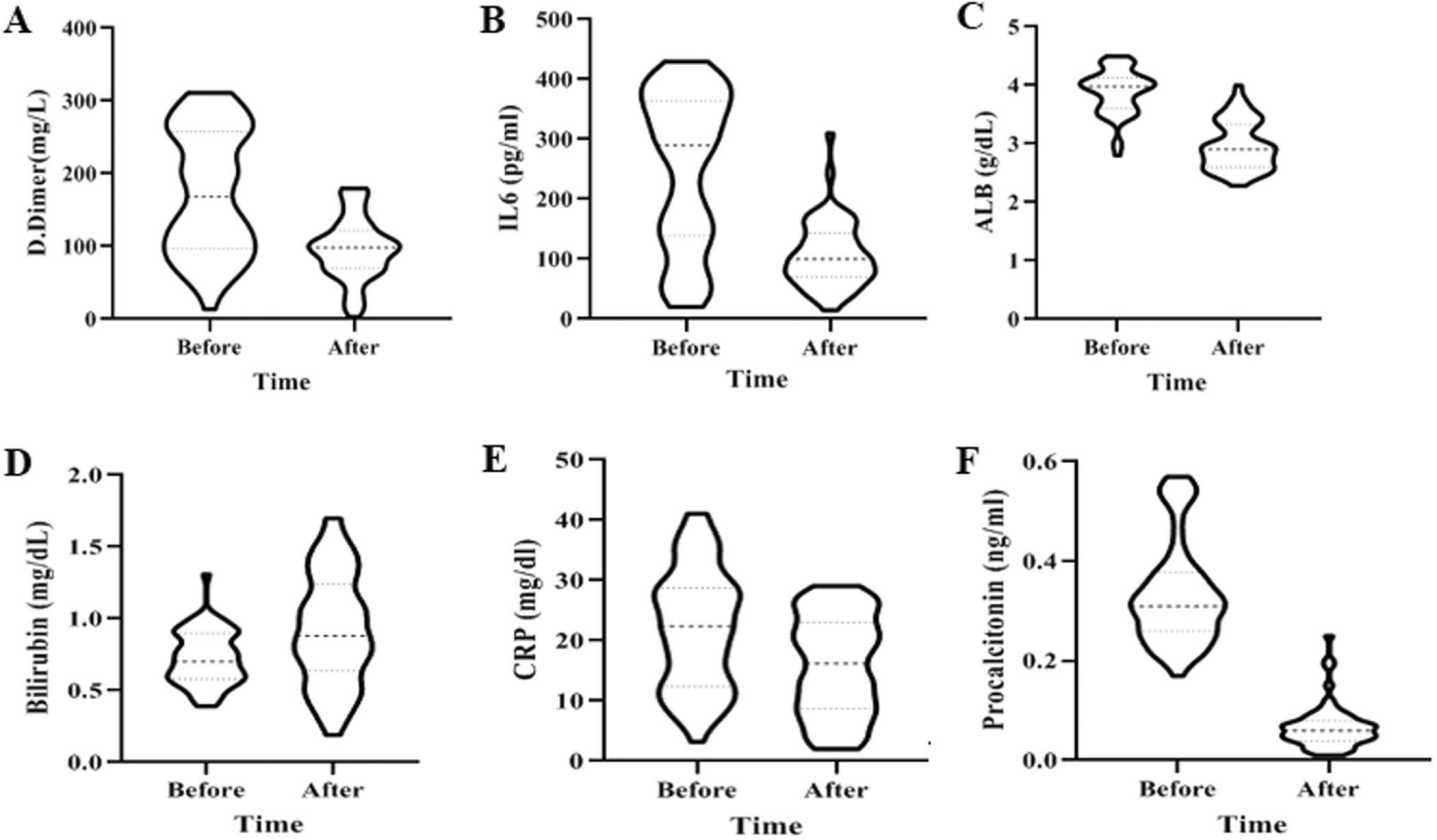
Violin plots for the comparison of (A) D.Dimer, (B) IL6, (C) ALB, (D) Bilirubin, (E) CRP, and (F) Procalcitonin pre and post CRRT. *p* = 0.042 for D.Dimer; *p* = 0.037 for IL‐6; *p* = 0.029 for ALB, *p* = 0.044 for Bilirubin; *p* = 0.025 for CRP and *p* < 0.01 for Procalcitonin.

The violin plots provide information about the distribution, variability, and central tendency of d‐Dimer, IL‐6, ALB, CRP, bilirubin, and PCL levels in the patient population (Figure 1).

Table 2 shows the comparison results of laboratory tests before and after HP/CRRT in patients with COVID‐19. Except for creatinine, all indicators have changed significantly compared to before HP/CRRT (*p* < 0.05). The SOFA score notably decreased from 7.6 ± 1.2 to 5.1 ± 1.0 (*p* < 0.05).

**TABLE 2:** Laboratory characteristics before and after HP/CRRT.

Items	Before	After	*p*‐value	Cohen *d*
Creatinine (mg/dL)	1.5 ± 1.4	1.4 ± 1.0	0.38	0.101
D.Dimer (mg/L)	179.9 ± 130.1	108.9 ± 75.7	0.04*	0.546
IL6 (pg/mL)	241.7 ± 192.0	105.9 ± 80.1	0.04*	0.676
ALB (g/dL)	3.9 ± 0.5	2.9 ± 0.5	0.03*	0.652
Bilirubin (mg/dL)	0.7 ± 0.2	0.9 ± 0.6	0.04*	0.698
CRP (mg/dL)	22.0 ± 18.8	16.3 ± 12.3	0.03*	0.466
Procalcitonin (ng/mL)	0.33 ± 0.25	0.07 ± 0.05	< 0.001*	0.668
Lactate level, mmol/L	4.7 ± 0.1	3.2 ± 0.1	< 0.001*	0.499
NGAL (ng/mL)	184.8 ± 160.5	154.6 ± 130.1	0.02*	0.644
SOFA	7.6 ± 1.2	5.1 ± 1.0	0.03*	0.507

* Significant at 0.05 level.

Table 3 shows the follow‐up data of patients at four time points. The changes in WBC, MAP, and Sao2 are not significant. On the other hand, variables such as Lymphocyte, LDH, PTT, and Ferritine had significant changes at the 5% level (*p* < 0.05).

**TABLE 3:** Follow‐up data.

Items	Before	During	After 24	After 48	*p*‐value	Cohen *d*
WBC	13.3 ± 5.4	13.6 ± 6.0	13.6 ± 6.3	14.2 ± 5.7	0.66	0.063
Hb	13.3 ± 2.1	13.0 ± 2.2	13.6 ± 2.3	13.7 ± 2.3	0.09**	0.201
Lymphocyte	515 ± 477.3	466.2 ± 420.0	470.5 ± 440.7	449.7 ± 398.0	< 0.001*	0.689
PLT	195.0 ± 60.2	199.3 ± 79.0	179.9 ± 81.0	170.7 ± 80.0	0.06**	0.199
LDH	1162.4 ± 582.8	1199.7 ± 553.9	1377.5 ± 703.5	1383.1 ± 493.9	0.04*	0.588
PTT	40.6 ± 21.4	48.3 ± 19.7	54.7 ± 29.9	56.0 ± 28.3	0.03*	0.698
INR	2.5 ± 0.7	2.0 ± 0.9	1.8 ± 1.0	1.6 ± 0.7	0.08**	0.234
Ferritin	1490.9 ± 715.3	1223.0 ± 685.1	666.0 ± 515.8	782.9 ± 588.3	0.03*	0.440
MAP	87.9 ± 7.9	88.1 ± 8.3	88.7 ± 7.5	89.0 ± 6.1	0.33	0.094
Sao2	85.4 ± 4.9	84.9 ± 5.0	85.3 ± 5.9	86.0 ± 4.7	0.11	0.201

* Significant at 0.05 level.

** Significant at 0.10 level.

**FIGURE 2:**
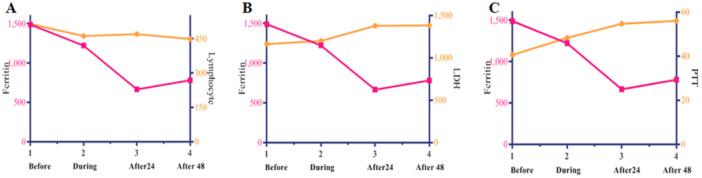
Comparison between ferritin and other variables with significant changes in follow up, including (A) Lymphocyte, (B) LDH, and (C) PTT. Ferritin and Lymphocyte were decreasing, while LDH and PTT were increasing. The simultaneous changes of these four variables can be seen in this graph. Pink color shows ferritin, and orange color is applied for the other three follow‐up variables.

As illustrated in Figure 2, ferritin and the Lymphocyte significantly decreased, while the levels of LDH and PTT increased.

## 4 Discussion

The COVID‐19 pandemic presented unprecedented challenges to the global community, resulting in devastating rates of disease and death as well as significant economic consequences. To combat this disease, extensive efforts have been devoted to finding effective treatments [20].

This study was conducted to investigate the efficacy of cytosorb hemoperfusion combined with CRRT for treatment of critically ill patients with COVID‐19 infection. Previously, we demonstrated a study using HP and CRRT on the mortality of COVID‐19 patients [21]. In the study, 12 patients with COVID‐19 and ARDS were divided into HP, CRRT, and HP + CRRT groups. Results showed that the combined HP + CRRT approach yielded the best outcomes, with a higher survival rate, improved oxygenation, and reduced inflammatory mediators. The CRRT group had the highest mortality rate, while HP alone did not show significant differences. These findings suggest that HP + CRRT may be an effective strategy for managing COVID‐19. However, one of the limitations of the study was a small sample size [21]. Therefore, the present study was designed with a larger sample size of 56 patients with COVID‐19 to compare various blood parameters before and after HP/CRRT.

Based on the results, all of the laboratory characteristics expect creatinine level changed significantly (Table 2), which, for better comparison, are illustrated in Figure 1. We observed a significant reduction in d‐Dimer levels after treatment. d‐dimer is commonly elevated in patients with COVID‐19 reflects both the inflammatory response and the heightened risk of thrombosis and hypercoagulability associated with the disease [22]. Reduction in d‐dimer levels following HP/CRRT signifies an improvement in blood clotting status.

Similarly, the levels of IL6 were significantly decreased. IL‐6 is a key mediator of inflammation, involved in both acute and chronic inflammatory processes [23]. It regulates immune cell recruitment and activation, induces the production of acute‐phase proteins, and contributes to tissue damage and remodeling [24, 25]. Numerous studies have consistently shown that increased plasma levels of cytokines, including IL‐1, IL‐6, and IL‐12 are strongly associated with severe lung inflammation in individuals with COVID‐19 pneumonia, leading to an increased risk of mortality [26, 27]. Patients with IL‐6 ≥ 80 pg/mL had a meaningfully higher probability of need of invasive MV. In the setting of COVID‐19, multiple organ failure and various life‐threatening complications, such as ARDS, heart damage, and renal and liver failure could be the result of the hyperinflammatory response [26]. Our findings, along with several other studies, have demonstrated that HP and CRRT exhibit remarkable efficacy in achieving high solute clearance rates using a semipermeable membrane. Furthermore, these therapeutic approaches have proven effective in eliminating inflammatory mediators from the bloodstream and decreasing systemic inflammation [21, 28].

Higher levels of serum CRP, an acute‐phase protein, indicate the presence of inflammation. In addition to IL‐6, CRP is a significant predictive marker for the severity of COVID‐19, as more severe patients with lung damage tend to have elevated levels of CRP [26]. Our results were consistent with previous studies that show a reduction in serum IL‐6 and CRP levels following HP [29] or CRRT in COVID‐19 patients [30]. On a positive note, considerable reductions in IL‐6, CRP, and d‐dimer levels were observed after HP and CRRT, suggesting a positive response to treatment and a reduction in systemic inflammation.

The present study also revealed that HP/CRRT therapy led to ALB increase and bilirubin decrease, associated with improved protein synthesis and liver function. Furthermore, we observed a significant decrease in the concentration of procalcitonin. Elevated PCT has been detected in COVID‐19 individuals, and PCT ≥ 0.56 ng/mL is related to higher mortality [31]. Noticeably reduction in PCT level following HP/CRRT indicated resolution or control of bacterial infections. Other interesting findings of our study were noteworthy decreasing SOFA score and lactate levels.

Numerous studies suggested level of lactate as a reliable biomarker of sepsis‐induced tissue hypoperfusion that is correlated with poor outcomes and mortality in critically ill patients with sepsis [32, 33]. A systematic literature review showed higher blood lactate levels in COVID‐19 patients with worse outcomes than in patients with better outcomes [34]. One study on 2860 patients with COVID‐19 indicated that elevated serum lactate is significantly associated with mortality [35].

NGAL is an important biomarker for acute kidney injury AKI and systemic inflammation, playing a crucial role in the early detection and management of kidney‐related complications in critically ill patients [36, 37]. NGAL is released in response to kidney injury, and elevated levels are indicative of renal stress. In this study, the significant decrease in NGAL levels from 184.8 ± 160.5 ng/mL to 154.6 ± 130.1 ng/mL after HP/CRRT highlights the potential efficacy of this combined therapy in mitigating renal injury and inflammation. The reduction in NGAL levels correlates with the overall improvement in inflammatory markers and suggests that HP/CRRT could be instrumental in addressing multiorgan dysfunction in severe COVID‐19 cases. This finding supports the utility of NGAL as a valuable marker for monitoring the therapeutic efficacy and renal recovery in such critical interventions.

Recently, Park et al. [38] inferred that the SOFA score after 24–48 h (ICU Days 2 or 3) might be a better predictor of in‐hospital mortality than the time zero of sepsis (ICU Day 1). They also showed that adding the Lactate score to the SOFA score (Lac‐SOFA score) more accurately predicted in‐hospital mortality than the SOFA score alone in sepsis patients [38]. In the present study, following HP/CRRT therapy, the SOFA score significantly decreased (from 7.6 ± 1.2 to 5.1 ± 1.0; *p* < 0.05), which is associated with lower organ failure severity. Moreover, the mortality rate was assessed at 41%, and the level of lactate reduced from 4.7 ± 0.1 to 3.2 ± 0.1 mM in treated patients.

Our study outcome contrast to the recent trial by Hutahaean et al. [39], which examined the use of HP therapy in severely ill COVID‐19 patients. In this study, majority of patients did not show improvement in their clinical and laboratory outcomes, including neutrophil‐lymphocyte ratio (NLR), ferritin, d‐dimer, and CRP values, after receiving 2–4 sessions HP using the MG150® cartridge. Additionally, they observed a high mortality rate among intubated patients with respiratory failure.

We summarized follow‐up data on various laboratory markers and clinical variables before, during, and after treatment of patients with severe COVID‐19 in Table 3. According to the results from the hematologic laboratory, SaO2 improved after HP; however, the improvement reduction was not statistically significant. There were also no significant differences in INR, WBC count and hemoglobin concentration before and after hemoperfusion.

On the other hand, the changes observed in ferritin, lymphocyte count, LDH, and PTT in COVID‐19 patients after CRRT provide important insights into the patients' immune response, organ function, and coagulation profile. Ferritin is an acute‐phase reactant and a marker of inflammation [40]. The decreasing trend in ferritin levels after HP/CRRT suggests a reduction in systemic inflammation. Lower ferritin levels may indicate a better control of the inflammatory response and improvement in the patients' condition.

In COVID‐19, lymphocyte depletion is commonly observed [41]. A notable observation was a significant decrease (*p* < 0.05) in lymphocyte count during and after HP/CRRT, which can indicate impaired immune function. In addition, a significant increase in LDH levels after 24 and 48 h suggests ongoing tissue damage or metabolic stress in some patients (Figure 2). Growing studies showed the positive relation between LDH level and mortality rate in COVID‐19 patients. However, LDH is a nonspecific marker that is found in various body tissues; additional clinical evaluation is required to understand the underlying causes of its elevation. There was also a significant increase in PTT after the specified time intervals compared to before. Prolonged PTT can indicate disturbances in blood coagulation pathways, potentially reflecting an increased risk of bleeding.

The effectiveness of hemoperfusion with a neutro‐macroporous resin device in sepsis patients has been reported. A considerable reduction of circulating cytokines appears to have positive benefits [42]. COVID‐19 individuals may have superimposed sepsis [43]. For this reason, HP is also employed as a therapy option in COVID‐19 patients, and numerous studies have noted its beneficial effects. Our study is consistent with previous studies. Recently, Surasit and Srisawat [44] have showed that the addition of early HA‐330 hemoperfusion to standard therapy led to clinical improvement, as evidenced by decreased SOFA scores (from 4.3 ± 1.9 to 3.5 ± 0.99) and improved CXR RALE scores. The 28‐day mortality rate was significantly lower in the HP group compared to the control group [44]. One study demonstrated the effectiveness of combining HP with CRRT in halting the progression of ARDS, reducing the need for intubation, and lowering mortality rates [17]. Another study by De Rosa et al. [45], revealed that HP with polymyxin contributed to the recovery from organ failure and improvement in hemodynamics.

In summary, these results highlight the effectiveness of extracorporeal therapies by CRRT/HP in managing COVID‐19 patients that showing significant improvements in laboratory markers associated with inflammation and organ dysfunction. The findings suggest that this combined therapeutic approach holds promise as a potential intervention for severe COVID‐19 cases with multiorgan dysfunction, potentially leading to improved clinical outcomes.

A significant limitation of our study is the absence of a control group. Although including a control group would have strengthened the study's findings, it was not feasible due to ethical considerations in our setting. Future research should ideally include control groups for more robust comparisons.

## 5 Conclusion

Combined HP and CRRT hold promise as a potential intervention for severe COVID‐19 cases with multiple organ dysfunction, leading to improved clinical outcomes.

## Author Contributions

**Seyed MohammadReza Hashemian:** conceptualization, funding acquisition, project administration, data curation, supervision, investigation, validation, visualization. **Ameneh Jafari:** writing – original draft, writing – review and editing, investigation. **Batoul Khoundabi:** investigation, formal analysis, data curation, methodology, software. **Hamidreza Jamaati:** funding acquisition, visualization, validation. **Payam Rahimi:** methodology, investigation.

## Conflicts of Interest

The authors declare no conflicts of interest. The manuscript has been read and approved by all authors, who believe that the manuscript represents honest work.

## Transparency Statement

The lead author, Seyed MohammadReza Hashemian, Ameneh Jafari affirms that this manuscript is an honest, accurate, and transparent account of the study being reported; that no important aspects of the study have been omitted; and that any discrepancies from the study as planned (and, if relevant, registered) have been explained.

## Data Availability

The data that support the findings of this study are available on request from the corresponding author. The data are not publicly available due to privacy or ethical restrictions.
